# Effects of Warming and N Deposition on the Physiological Performances of *Leymus secalinus* in Alpine Meadow of Qinghai-Tibetan Plateau

**DOI:** 10.3389/fpls.2019.01804

**Published:** 2020-02-21

**Authors:** Hao Shen, Shikui Dong, Shuai Li, Wenying Wang, Jiannan Xiao, Mingyue Yang, Jing Zhang, Xiaoxia Gao, Yudan Xu, Yangliu Zhi, Shiliang Liu, Quanming Dong, Huakun Zhou, Jane C. Yeomans

**Affiliations:** ^1^ School of Environment, State Key Joint Laboratory of Environmental Simulation and Pollution Control, Beijing Normal University, Beijing, China; ^2^ School of Life and Geographic Sciences, Qinghai Normal University, Xining, China; ^3^ Qinghai Academy of Animal Husbandry and Veterinary Science, Qinghai University, Xining, China; ^4^ Northwest Institute of Plateau Biology, Key Laboratory of Restoration Ecology of Cold Are in Qinghai Province, Chinese Academy of Science, Xining, China; ^5^ Research Department, Earth University, San José, Costa Rica

**Keywords:** Qinghai-Tibetan plateau, stomatal conductance, antioxidant enzymes, photosynthesis, *Leymus secalinus*

## Abstract

Warming and Nitrogen (N) deposition are key global changes that may affect eco-physiological process of territorial plants. In this paper, we examined the effects of warming, N deposition, and their combination effect on the physiological performances of *Leymus secalinus*. Four treatments were established in an alpine meadow of Qinghai-Tibetan plateau: control (CK), warming (W), N deposition (N), and warming plus N deposition (NW). Warming significantly decreased the photosynthetic rate (*A_net_*), stomatal conductance (*g_s_*), intercellular CO_2_ concentration (*C_i_*), and transpiration rate (*T_r_*), while N deposition and warming plus N deposition significantly increased those parameters of *L. secalinus*. Warming significantly increased the *VPD* and *L_s_*, while N deposition and warming plus N deposition had a significant positive effect. Warming negatively reduced the leaf N content, Chla, Chlb, and total Chl content, while N deposition significantly promoted these traits. Warming, N deposition, and their combination significantly increased the activity of SOD, POD, and CAT. Besides, warming and warming plus N deposition significantly increased the MDA content, while N deposition significantly decreased the MDA content. N deposition and warming plus N deposition significantly increased the Rubisco activity, while warming showed no significant effect on Rubisco activity. N deposition and warming plus N deposition significantly increased the *Fv*/*Fm*, ΦPSII, *qP*, and decreased NPQ, while warming significantly decreased the *Fv*/*Fm*, ΦPSII, *qP*, and increased NPQ. N deposition strengthened the relations between *g_s_*, Chl, Chla, Chlb, Rubisco activity, and *A_net_*. Under warming, only *g_s_* showed a significantly positive relation with *A_net_*. Our findings suggested that warming could impair the photosynthetic potential of *L. secalinus* enhanced by N deposition. Additionally, the combination of warming and N deposition still tend to lead positive effects on *L. secalinus*.

## Introduction

Climate warming and Nitrogen (N) deposition, as two major factors that drive global change, are predicted to increase simultaneously in the future (Zong et al., 2018a). The global surface temperature has been significantly increased since the industrial revolution and predicted to increase by approximately 0.9°C-5.4°C in this century (IPCC, 2013). Grassland, the dominant ecosystem which cover over 60% of local territory (Dong and Sherman, 2015), is mainly distributed in semi-dry or dry areas in the QTP (Qin et al., 2014 **) and is very vulnerable to global changes (Chen et al., 2014).

Climate warming has been considered as one of the highly influential factors that pose a threat to ecological integrity and function (Hughes et al., 2018). It can influence carbon sequestration in terrestrial ecosystems, especially alpine ecosystems (Ganjurjav et al., 2018). Warming and drying over the QTP is becoming serious for decades (Chen et al., 2014), which may affect plant physiological processes such as photosynthesis and enzymatic antioxidant (Shi et al., 2010; Yu et al., 2019). Generally, photosynthetic process and the antioxidant enzyme are the most sensitive to climate warming (Atkin et al., 2006). A recent study has reported that warming can significantly decrease plant stomatal conductance on the QTP (Zhang et al., 2010), thus affecting plant photosynthesis. Until now, warming effects on plant photosynthesis remain controversial (Niu et al., 2008); e.g., positive (Shaw et al., 2000), neutral (Loik et al., 2000), and negative (He and Dong, 2003) effects have been reported by previous researchers. Warming can increase the temperature of air and soil, and reduce soil water content (Allison et al., 2018; Samaniego et al., 2018), finally leading to dramatic ecological effects on plants. Previous study found that warming-induced increase of soil temperature and decrease of soil moisture can lead to declined biomass accumulation in herbaceous perennial plants (Harte and Shaw, 1995). Besides, plant water potential may be impaired under warming by a reduction in stomatal conductance and photosynthetic CO_2_ assimilation, which leads to a lower photosynthetic rate and more non-photochemical quenching (Loik et al., 2000). Additionally, warming-induced water stress can lead to photoprotection of PSII, which is important for plant adaptation (Demmig-Adams and Adams, 1996), as downregulation of PSII reaction centers and non-photochemical quenching under environmental stress is of high significance (Valladares and Pearcy, 1997). Many efforts have been made to examine the effects of climate warming on growth and physiological process of temperate plants (Nakaji et al., 2001), yet little is known about the effects of climate warming on plant physiology of alpine grassland. Plant physiology is highly sensitive to temperature, a slight change in temperature may lead to a significant alteration of plant physiological process (Atkin et al., 2006). Therefore, a better understanding of ecosystem responses to warming from the perspective of plant physiological acclimation is critically important.

N deposition is another important component of global change. Though high latitude regions are usually N-limited (Marañón-Jiménez et al., 2019), the rate of N deposition on Qinghai-Tibetan plateau has significantly increased in recent decades (Zhang et al., 2018a). However, the influences of N deposition on plant performance are still unclear. Photosynthesis is strongly influenced by N availability (Wang et al., 2018), e.g., change of leaf N content can alter the ribulose-1, 5-bisphosphate carboxylase/oxygenase (Rubisco) activity and thus affect plant photosynthesis (Cheng and Fuchigami, 2000). N deposition could regulate plant responses to warming (Zong et al., 2018b), and change in temperature is also likely to interact with the effects of N deposition (Suddick et al., 2013). Up to now, little is known about the physiological responses of plants under warming and N deposition.

*Leymus secalinus* is one of the key grass species in alpine grasslands of the QTP (Mou et al., 2018). Through field investigation in our study site, we found that *L. secalinus* is the most dominant grass species accounting for nearly 50% of the total coverage under each treatment. Therefore, we choose this species as our model plant to investigate the effects of warming and N deposition on plants. We assumed that climate warming and N deposition can alter photosynthetic potential of *L. secalinus* through changing leaf nutrients, gas exchange, photosynthetic enzyme, antioxidant metabolism, and chlorophyll fluorescence. To test this hypothesis, we conducted this study with the objective of disclosing the possible adaptable mechanism in physiology of *L. secalinus* under warming and N deposition. Our objectives were to: 1) determine the physiological performances of *L. secalinus* response to warming and N deposition 2) explore main factors that determine the alteration of *L. secalinus* photosynthesis under warming and N deposition 3) disclose the direct and indirect path that lead to the variation of *A_net_* under warming and N deposition. The study is helpful to better understand the latent mechanisms of adaptation of alpine grassland plants to N deposition and climate warming in the future.

## Material and Methods

### Study Sites

**Figure 1:**
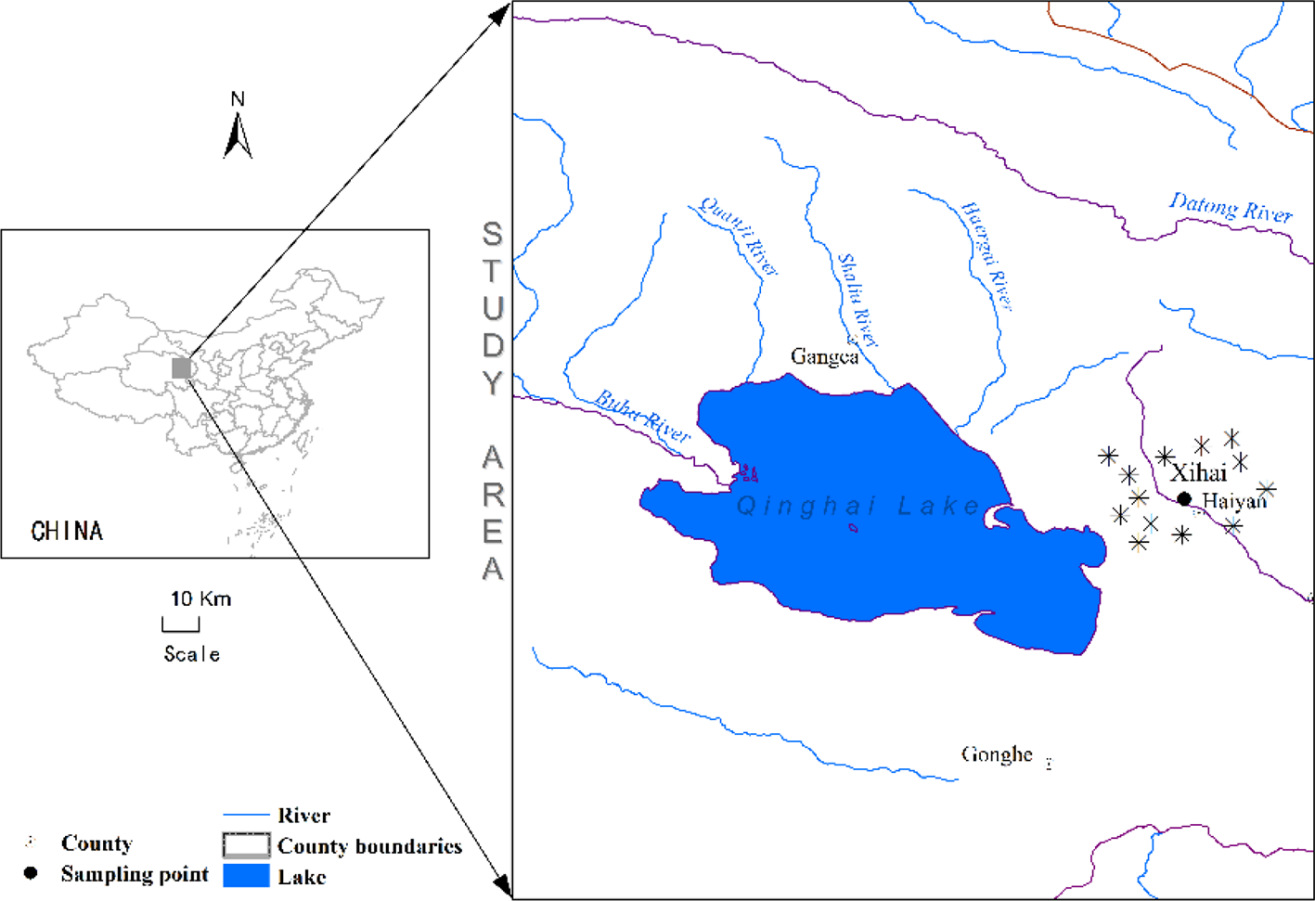
Location of study site.

This study was conducted in Xihai Town, Haiyan County (100°57′E, 36°56′N, 3100 m ASL), which is located at the eastern side of Qinghai Lake with mean annual temperature about 1.4°C, mean annual precipitation about 330-370 mm, and annual evaporation capacity around 1400 mm. The soil was mostly loam-clay (Zhao et al., 2017) (**Figure 1**).

### Experimental Design

A complete randomized block factorial experimental design was used. Four treatments: control treatment (CK), warming alone (W), N deposition alone (N), and warming plus N deposition (NW) were established for the experiment in this study. In early 2015, we fenced twelve 2 m × 5 m plots in an alpine meadow of Qinghai-Tibetan plateau. Three plots (replicates) were randomly selected for the control treatments; three plots (replicates) were randomly selected and treated with ammonium nitrate (NH_4_NO_3_) of 8 kg N ha^−^^1^ year^−^^1^, which is the annual N deposition of this area (Lü and Tian, 2007); and another three plots (replicates) were randomly selected for the warming treatment by placing the open top chambers (OTC), in which the mean temperature in July (growing season) was about 2°C higher than the outside (**Table 1**). The remaining three plots were randomly selected for warming plus N deposition by placing the open-top chambers (OTC) and fertilizing with 8 kg N ha^−^^1^ year^−^^1^ NH_4_NO_3_. The interval between plots was 1 m. All the plots were similar in the topographies and land use histories. The treatments of warming and nitrogen application treatments were initiated since early May of 2015, and the corresponding plots were fertilized with ammonium nitrate (NH_4_NO_3_) in early May and July every year. Soil temperature and moisture were measured using digital temperature sensors (**Table 1**).

**Table 1:** Means ( ± SE) of air temperature (°C) and soil temperature (°C) in July 2018 in the study plots.

Treatment	Air temperature (°C)	Soil temperature (°C)
**CK**	16.38 ± 0.24b	17.18 ± 0.39b
**W**	18.41 ± 0.11a	19.53 ± 0.20a

CK represents no warming and no N deposition; W includes warming and warming plus N deposition. Different lowercase letters indicate significant differences between treatments in the same sampling period (P < 0.05).

### Sample Collection

Plant samples were collected in July 2018 (growing season). Nine *L. secalinus* with similar growth status were chosen, and the completely expanded and intact leaves of each plant were used for gas exchange and chlorophyll fluorescence measurement, then the leaves were wrapped in aluminum foil, immediately immersed in liquid nitrogen, and stored at -80°C for laboratory analysis.

### MDA Content Measurement

The malondialdehyde (MDA) content was measured according to the method of thiobarbituric acid (TBA) (Heath and Packer, 1965) with some modifications. The liquid nitrogen-stored leaves of *L. secalinus* were stored thawed and 0.1 g of fresh leaves were ground in a ceramic mortar. Liquid nitrogen and 5 ml 10% trichloroacetic acid (TCA) were added into the mortar until the formation of a homogeneous powder. The homogenate was centrifuged at 4000 × g for 15 min at 4°C, then 2 ml supernatant was mixed with 5 ml of 0.6% TBA in 10% TCA and heated for 15 min, cooled in an ice bath to room temperature, and centrifuged once again. Finally, the absorbance was measured at 450, 532, and 600 nm. The MDA content was measured according to the below formula: MDA (μmol·L^-1^) = 6.45 (A_532_-A_600_) - 0.56A_450_, and then expressed as μmol·g^−1^ fresh weight.

### Chlorophyll Extraction

The chlorophyll and carotenoid contents in the *L. secalinus* leaves of each treatment were extracted with 80% acetone according to Lichtenthaler (1987). The absorbance of chlorophyll a (Chla) and chlorophyll b (Chlb) was measured at 663 nm and 646 nm, respectively, and their concentrations (mg·g^-1^ FW) were calculated according to the following equations:

$$\begin{array}{l} \text{Chla}=12.21{\text{A}}_{663}-2.81{\text{A}}_{646}\\ \text{Chlb}=20.13{\text{A}}_{646}-5.03{\text{A}}_{663}\\ \text{Total Chl}=\text{Chla}+\text{Chlb} \end{array}$$

### Gas Exchange Parameters Measurement

Fully expanded and exposed leaves of selected samples were measured between 9:00 and 11:00 (local time) in each plot on sunny days using a portable photosynthesis system (*Li6400XT*, *Li-Cor*, *Inc*., Lincoln, NE, USA). Three individual plants in a similar healthy state per replicate were selected for sampling. Three intact leaves of each individual were measured for statistical analysis. The photon flux density (PFD) was maintained at 1500 μmol·m^-2^·s^-1^ using the red-blue source of *Li6400XT*. In the chamber, the temperature was maintained at 25°C, with a relative humidity of 55%-70%. The CO_2_ concentration was maintained at 400 μmol·mol^-1^. The net photosynthetic rate (*A_net_*), stomatal conductance (*g_s_*), intercellular CO_2_ concentration (*C_i_*), transpiration rate (*T_r_*), vapor pressure deficit (*VPD*), and stomatal limitation (*L_s_*) were measured as *L_s__=_* 1-*C_i__/_C_a_* by following Yin et al.’s (2006) methods.

### Chlorophyll Fluorescence Measurement

After the measurements of gas exchange parameters, the marked leaves were wrapped in tin foil paper for dark adaptation for 30 minutes before measurement (ensuring total darkness) in order to standardize chlorophyll activity of all leaves. Then, it was measured with a portable *LI-6400-40* pulse-amplitude-modulation fluorometer (*Li-cor*, USA). The measured fluorescence variables included the following: maximum photochemical efficiency of PSII (Fv/Fm), actual photochemical efficiency of PSII (ΦPSII), photochemical quenching coefficient (qP), and non-photochemical quenching coefficient (NPQ). Light-adapted components of chlorophyll fluorescence, including steady-state yield (*F*) and maximum fluorescence yield (*Fm′*), were measured and calculated as ΦPSII = *Fm'*–*F*/*Fm'* (Genty et al., 1989); dark-adapted components of chlorophyll fluorescence minimum fluorescence yield (*Fo*) and maximum fluorescence yield (*Fm*) were measured and calculated as *Fv*/*Fm =* (*Fm*–*Fo*)/*Fm* (Fleck et al., 1998), qP = (*Fm'*–*F*)/(*Fm'*–*Fo'*) (Oxborough and Baker, 1997); and non-photochemical quenching was calculated as NPQ = (*Fm*/*Fm′*) ^–1^(Barker and Adams, 1997).

### Antioxidant Enzymes Activities Measurement

Fresh leaf samples (0.4g) were ground in liquid nitrogen using a mortar and pestle, then the ground samples were homogenized under ice-cold conditions in 4.5 mL of the extraction buffer (0.1 M phosphate buffer containing 0.5 mM ethylenediaminetetraacetic acid, pH 7.4). Then, the homogenate was centrifuged at 8000 ×g for 15 min at 4°C. The supernatant was stored at -70°C for the analysis of antioxidant enzymes. SOD activity was measured by the percentage reduction of nitroblue tetrazolium (NBT) at 560 nm (Giannopolitis and Ries, 1977). One unit of SOD activity was defined as the amount of enzyme that leads to 50% inhibition of NBT reduction. Guaiacol oxidation by H_2_O_2_ at 420 nm was measured for the POD activity (Kochhar et al., 1979). The reaction mixture contained 2.4 mL of 0.1 M potassium phosphate buffer (pH 7.4), 0.3 mL 2% H_2_O_2_, 100 µL of the enzyme extract, and 1 mL of 50 mM guaiacol. Catalase (CAT) activity was measured according to the consumption of H_2_O_2_ at 240 nm (Trevor et al., 1994). One unit of CAT activity was defined as the amount of enzyme catalyzing the decomposition of 1 nmol of H_2_O_2_ per min. The reaction mixture contained 1 mL of 0.1 M potassium phosphate buffer (pH 7.4), 50 µL of the enzyme extract, and 0.1 mL of 0.1 M H_2_O_2_.

### Rubisco Activity Measurement

When measuring Rubisco activity, 0.1 g frozen leaf tissues was ground to a fine powder in mortar with liquid nitrogen according to Hu et al. (2010). 6 ml cooling extraction buffer was added, containing 50 mM Hepe-KOH (pH 7.5), 10 mM DTT, 2 mM EDTA, 10% glycerin(w/v), 1% BSA (w/v), 1% TritonX-100 (v/v), and 1.5% PVPP (w/v). Then, 1 ml homogenate was centrifuged at 14000 g for 10 min at 4°C. The supernatant was taken for Rubisco activity measurement in the 900 μl reaction medium containing 100 mM bicine (pH 8.0 at 25°C), 25 mM KHCO_3_, 20 mM MgCl_2_, 3.5 mM ATP, 5 mM phosphocreatine, 5 units glyceraldehyde-3-phosphate dehydrogenase, 5 units 3-phosphoglyceric phosphokinase, and 17.5 units creatine phosphokinase and 0.25 mM NADH. We added 50 μl enzyme extracting solution and let the solution rest for 15 min at 25°C, then added 50 μl Rubp, and recorded the absorbance every 10 s for 2 min at 340 nm.

### Statistical Analysis

All data were pooled in Excel 2016. Statistical analyses of data were performed using SPSS 22.0. Analysis of variance (ANOVA) was used to detect the effects of warming, N deposition, and their interactions. *Post hoc* comparisons were performed using Duncan's multiple tests at a significance level of *P* < 0.05. R package networkD3 was used to draw the relations. Structural equation modeling (SEM) was performed to identify the direct and indirect pathways that may affect photosynthesis of *L. secalinus* in response to warming and N deposition, which was performed by AMOS 22.0 (Amos Development, Spring House, PA, USA).

## Results

### Warming and N Deposition Lead to Different Effects on the Gas Exchange

**Figure 2:**
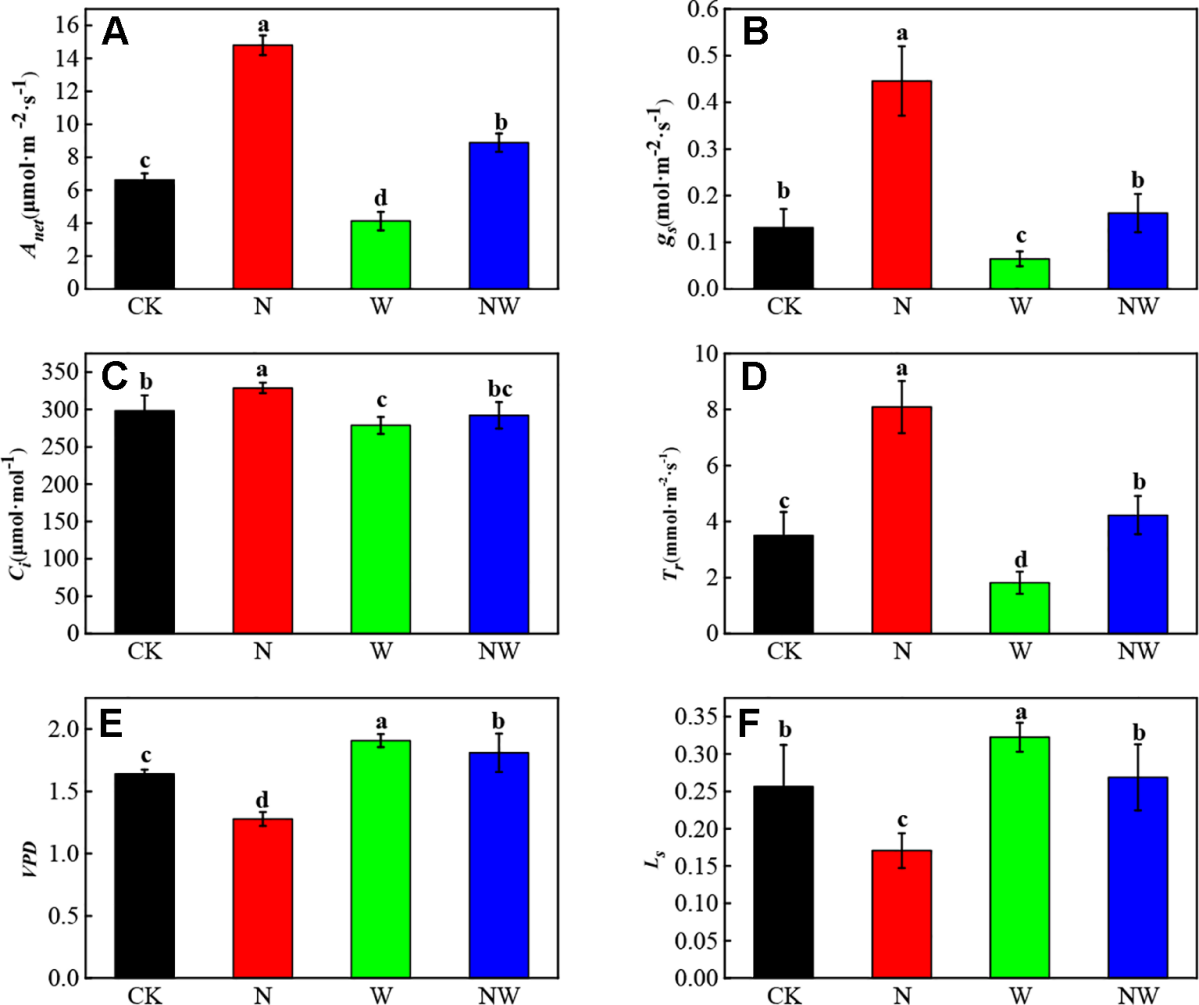
Effects of warming and N deposition on gas exchange parameters of *L. secalinus*. Vertical bars represent ± SE of the mean, and different letters on the SE bars indicate significant differences among all treatments (*P* < 0.05). **(A)** net photosynthetic rate **(B)** stomatal conductance **(C)** intercellular CO_2_ concentration, **(D)** transpiration rate **(E)** vapor pressure deficit **(F)** stomatal limitation value. Each treatment has three replicated plots, and nine leaves from nine *L. secalinus* with similar growth status were chosen in each plot as a biological replication for analysis (3 biological replicates were performed).

There existed a significant difference of gas exchange parameters under warming and N deposition (**Figure 2**). Compared with the CK, N and NW treatment significantly (*P* < 0.05) increased *A_net_* of *L. secalinus*, while W treatment significantly (*P* < 0.05) decreased *A_net_* of *L. secalinus*. The *A_net_* of *Leymus secalinus* under NW treatment was lower than that under N treatment alone (*P* < 0.05, **Figure 2A**). Similarly, the change of *g_s_, C_i_,* and *T_r_* showed a same dynamic with the *A_net_* under W and N treatment (**Figures 2A–D**). The *VPD* significantly increased in W treatment and decreased in N treatment (**Figure 2E**). Besides, the *L_s_* showed an opposite variation with *g_s_* under W and N treatment (**Figures 2B, D**).

### Warming and N Deposition Change in the Leaf N and Chlorophyll Content

**Figure 3:**
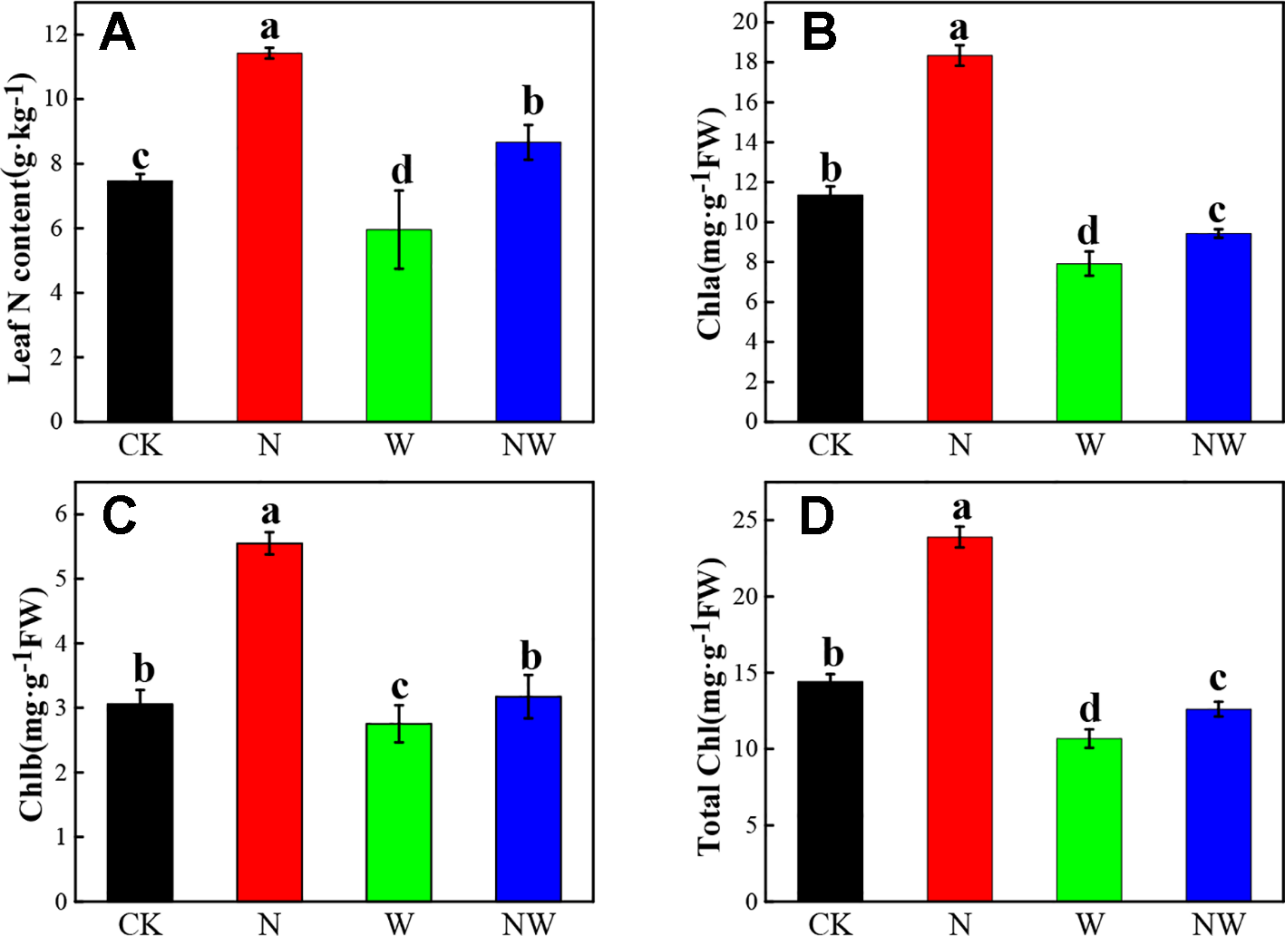
Effect of warming and N deposition on Leaf N and chlorophyll content of *L. secalinus*. Vertical bars represent ± SE of the mean, and different letters on the SE bars indicate significant differences among all treatments (*P* < 0.05). **(A)** Leaf N content **(B)** Chla **(C)** Chlb **(D)** total Chl. Each treatment has three replicated plots, and nine leaves from nine *L. secalinus* with similar growth status were chosen in each plot as a biological replication for analysis (3 biological replicates were performed).

According to **Figure 3A**, leaf N content significantly increased in N and NW treatment, while it decreased in W treatment compared with the CK (*P* < 0.05). The leaf chlorophyll content showed a similar variation with leaf N content under all treatments (**Figures 3B–D**).

### Warming and N Deposition Change in the Antioxidant System

**Figure 4:**
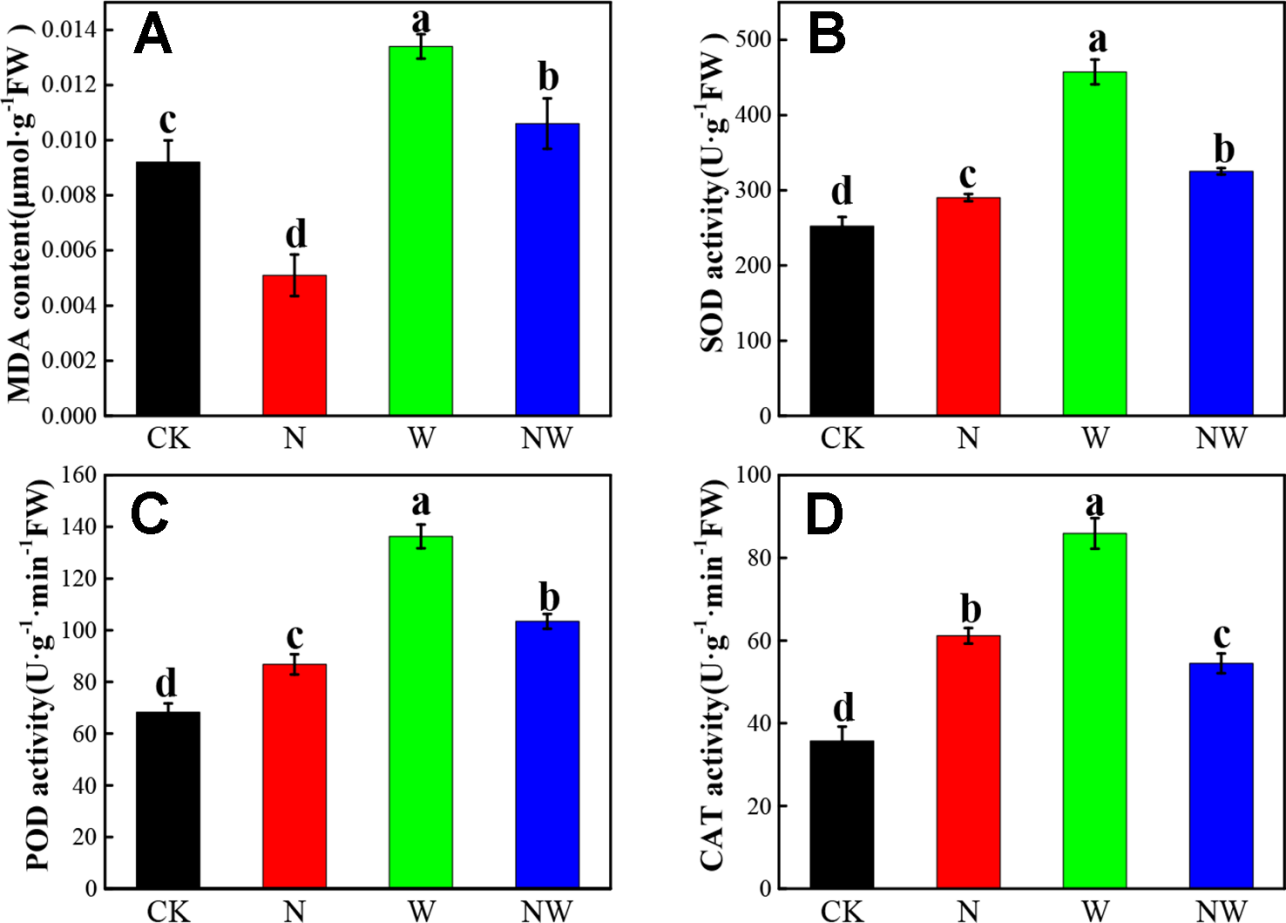
Effect of warming and N deposition on the MDA and antioxidant enzyme activity of *L. secalinus*. Vertical bars represent ± SE of the mean, and different letters on the SE bars indicate significant differences among all treatments (*P* < 0.05). **(A)** MDA content **(B)** SOD activity **(C)** POD activity **(D)** CAT activity. Each treatment has three replicated plots, and nine leaves from nine *L. secalinus* with similar growth status were chosen in each plot as a biological replication for analysis (3 biological replicates were performed).

The MDA content of *L. secalinus* significantly decreased in the N treatment, while it increased in W and NW treatment (*P* < 0.05) (**Figure 4A**). Additionally, the MDA content of *L. secalinus* under N and NW treatment was significantly (*P* < 0.05) lower than that under W treatment alone. N, W and NW treatment significantly enhanced the SOD, POD, and CAT activity (*P* < 0.05), and the activity of three antioxidant enzymes under W treatment were highest among all the treatments (**Figures 4B–D**).

### Warming and N Deposition Lead to Differential Variation of Photosynthetic Enzyme

**Figure 5:**
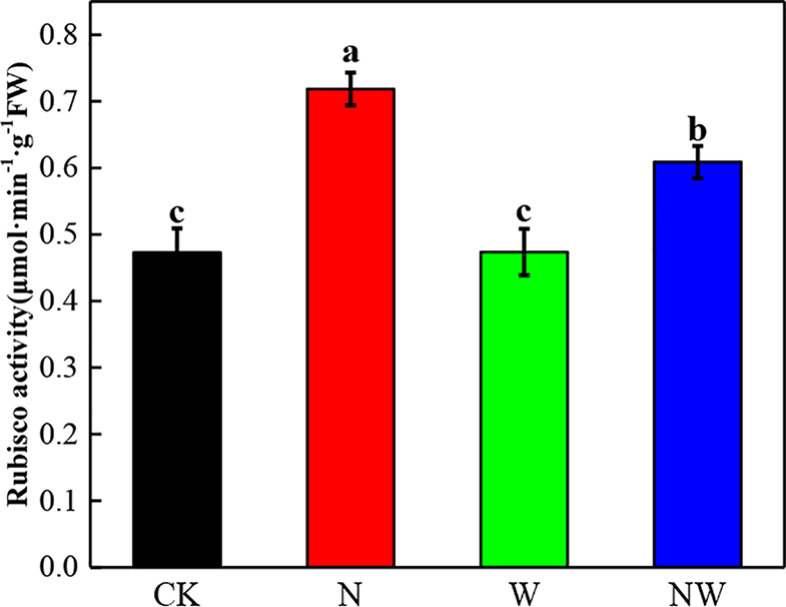
Effect of warming and N deposition on Rubisco activity of *L. secalinus*. Vertical bars represent ± SE of the mean, and different letters on the SE bars indicate significant differences among all treatments (*P* < 0.05). Each treatment has three replicated plots, and nine leaves from nine *L. secalinus* with similar growth status were chosen in each plot as a biological replication for analysis (3 biological replicates were performed).

N and NW treatment significantly (*P* < 0.05) increased the Rubisco activity of *L. secalinus*. W treatment had no significant (*P* > 0.05) influence (**Figure 5**).

### Warming and N Deposition Lead to Obvious Variation of Chlorophyll Fluorescence

**Figure 6:**
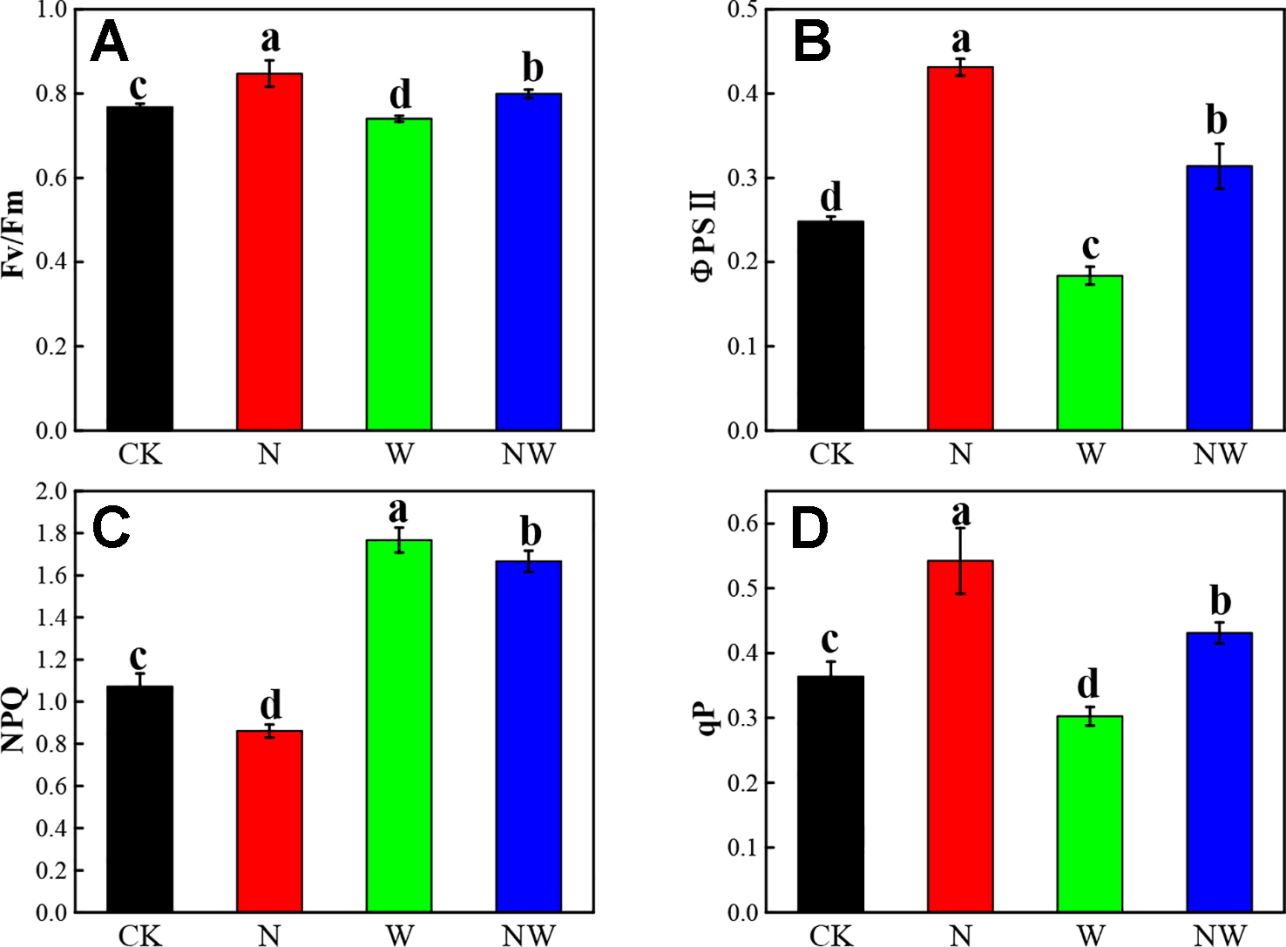
Effect of warming and N deposition on chlorophyll fluorescence of *L. secalinus*. Vertical bars represent ± SE of the mean, and different letters on the SE bars indicate significant differences among all treatments (*P* < 0.05). **(A)** Fv/Fm **(B)** ΦPSII **(C)** NPQ **(D)** qP. Each treatment has three replicated plots, and nine leaves from nine *L. secalinus* with similar growth status were chosen in each plot as a biological replication for analysis (3 biological replicates were performed).

The *Fv*/*Fm*, ΦPSII, and *qP* of *L. secalinus* showed a similar variation in N and W treatment (**Figures 6A, B, D**). N and NW treatment significantly increased the *Fv*/*Fm*, ΦPSII, and *qP* of *L. secalinus*, while W treatment significantly decreased theses three parameters (*P* < 0.05). N treatment significantly decreased the NPQ, while W and NW treatment significantly increased that index (**Figure 6C**) (*P* < 0.05).

### Main Factors Affect *A_net_* Under Warming and N Deposition

**Figure 7:**
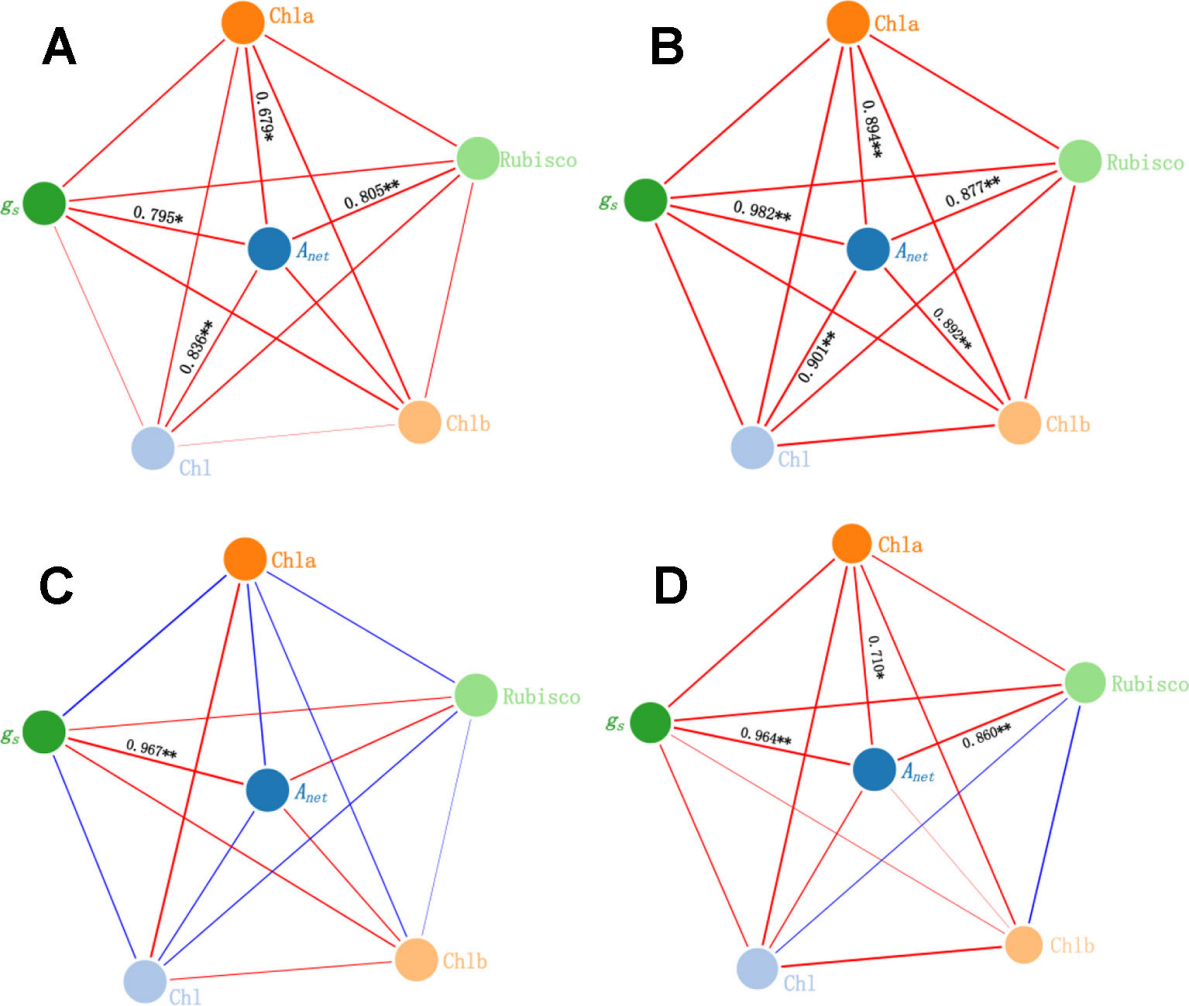
Relationship among *g_s_*, Chl, and Rubisco activity and *A_net_* under warming and N deposition. Red lines represent positive relations, while blue lines represent negative relations. The boldness of the lines indicates the strength of the relations. Asterisk (*P < 0.05, **P < 0.01) represents significant difference. Four treatments: **(A)** CK**, (B)** N**, (C)** W**, (D)** NW.

In the CK, *g_s_*, Chl, Chla, and Rubisco activity were positively correlated with *A_net_* (*r =* 0.795, *P* < 0.05; *r =* 0.836, *P* < 0.01; *r =* 0.679, *P* < 0.05, and *r =* 0.805, *P* < 0.01, respectively) (**Figure 7A**). Under the N alone treatment, *g_s_*, Chl, Chla, Chlb, and Rubisco activity were positively correlated with *A_net_* (*r =* 0.982, *P* < 0.01; *r =* 0.901, *P* < 0.01; *r =* 0.894, *P* < 0.01; *r =* 0.892, *P* < 0.01; and *r =* 0.877, *P* < 0.01, respectively) (**Figure 7B**). Under the warming treatment alone, only *g_s_* was positively correlated with *A_net_* (*r =* 0.967, *P* < 0.01) (**Figure 7C**). Under the warming treatment plus N deposition, *g_s_*, Chla, and Rubisco activity were positively correlated with *A_net_* (*r =* 0.964, *P* < 0.01; *r =* 0.710, *P* < 0.05; and *r =* 0.860, *P* < 0.01, respectively) (**Figure 7D**).

### Path Analysis Under Warming and N Deposition

**Figure 8:**
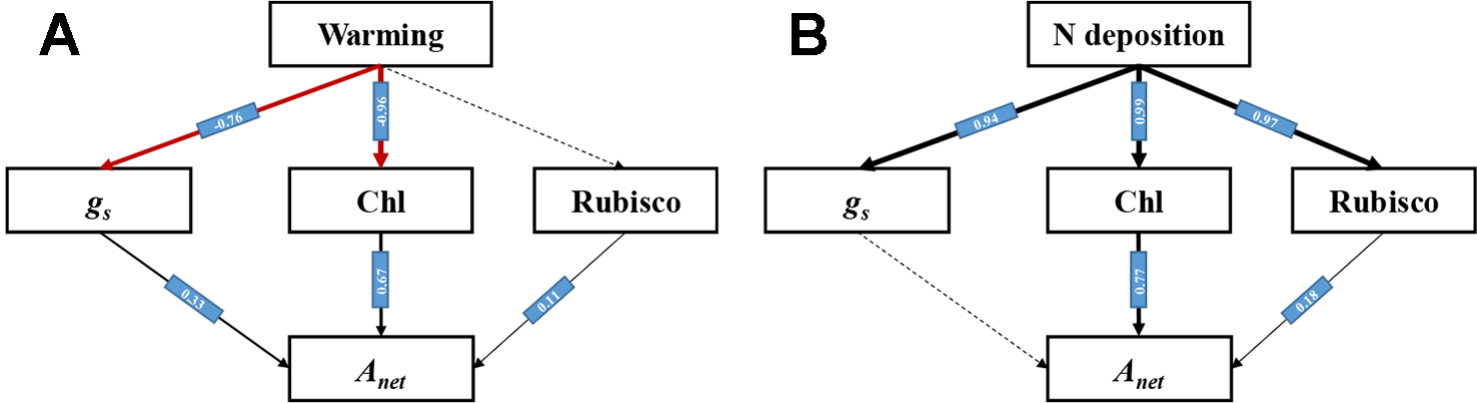
Path model (χ^2^ = 4.6, *P* > 0.05, CFI = 0.991) of warming and N deposition on net photosynthetic rate of *L. secalinus*. Black arrows with standardized coefficients show significant positive effects, while red arrows show significant negative effects (*P* < 0.05). Dash arrows show non-significant effects. **(A)** Path analysis under warming **(B)** Path analysis under N deposition.

A structural equation model (SEM) was successfully established. The paths under warming and N deposition were different (**Figure 8**).Warming caused the decrease of *A_net_* directly through reducing gs and leaf Chl content (*P* < 0.05), while it had no significant influence on Rubisco activity (*P* > 0.05) (**Figure 8A**). N deposition significantly increased the gs, leaf Chl content, and Rubisco activity (*P* < 0.05), while it mainly increased the *A_net_* through the enhancement of leaf Chl content and Rubisco activity (**Figure 8B**).

## Discussion

### N Deposition Has Positive Effects on Photosynthetic Capacity While Warming Causes Negative Effects

Warming can alleviate low temperature stress in the alpine region to some degree (Lu et al., 2013) and lead to water stress by increasing evapotranspiration as well (Zhang et al., 2018b). Positive effects of warming on gas exchange of some plant species have been reported (Loik et al., 2004), while a few researchers argued that temperature increase in the warm seasons may also limit plant gas exchange due to overheating (Llorens et al., 2004). In this study, we found that the net photosynthetic rate of *L. secalimus* significantly decreased under warming treatment, indicating that warming can negatively limit plant photosynthesis. We found that the soil moisture also significantly decreased under warming, suggesting that warming-induced decrease in soil moisture might be the main cause of photosynthesis inhabitation (Loik et al., 2000). Warming can also reduce the diffusion of CO_2_ to leaf leading to decreased stomatal conductance caused by an increase in vapor pressure deficit that usually occurs as temperature rises (Farquhar and Sharkey, 1982). Additionally, the stomatal conductance was significantly lower and stomatal limitation value was much higher under warming in our study, suggesting that warming can undermine plant photosynthesis through alteration of plant stomatal traits, such as decrease of stomatal density (Ferris et al., 1996). It is well known that plant stomatal conductance is an important factor affecting carbon gain and water use efficiency (Zheng et al., 2013), and it has been widely used to quantify the efficiency of gas exchange (Franks et al., 2009). In our study, warming caused the decline of *g_s_* and *C_i_*, indicating that stomatal limitation played a dominant role (Flexas and Medrano, 2002). However, under the warming plus N deposition treatment, the *g_s_* and *C_i_* were increased simultaneously, suggesting the predominance of non-stomatal limitation to photosynthesis, and N deposition was the main factor that drive the improvement of photosynthesis in QTP.

Additionally, the effects of warming on plants are always regulated by N deposition. Previous researchers have found that N deposition could affect plant gas exchange (Li et al., 2003). In agreement with (Zhao and Liu, 2008), we found that N deposition significantly induced a positive effect on *A_net_* of *L. secalimus*. Further, *A_net_* was significantly increased under combination of warming and N deposition, yet it was significantly decreased under warming alone, indicating N deposition may compensate for the warming-induced impact on photosynthesis. Under N deposition treatment, *A_net_*, *gs,* and *Ci* were significantly increased, suggesting that adequate supply of N can promote CO_2_ fixation.

The chlorophyll fluorescence reflects the basic function of the photosynthetic apparatus and the capacity and performance of photosynthesis (Zhao and Liu, 2008). Previous study showed that warming might promote plant photosynthesis through increasing apparent quantum yield (Awada et al., 2003). However, the results in our study showed that warming decreased the PSII efficiency of *L. secalimus* and induced photo-inhibition. Warming-induced soil drying could lead to a loss of excitation energy transfer and could impact the PSII efficiency (Shi et al., 2010), as PSII efficiency usually depends on temperature and water stress (Valentini et al., 1995). The Fv/Fm, ΦPSII, and qP values were significantly increased by N supply, suggesting that N was positively related to the enhanced efficiency of PSII center, as N deposition could significantly promote the synthesis of light-harvesting pigments, Chla and Chlb. The NPQ of *L. secalimus* significantly increased under warming, indicating that more energy participated in heat dissipation in order to alleviate oxidative stress (Shi et al., 2010). Additionally, our study on the Rubisco activity suggested that warming didn't cause enough or severe water stress to limit Rubisco activity. Rubisco wouldn't limit photosynthesis until severe or long-term water stress is encountered (Flexas et al., 2006).

### Warming and N Deposition Alter Leaf N Content, and Thus Influence the Synthesis of Chlorophyll

Leaf N content is always related to plant eco-physiological process that can reflect adaptation mechanism of the plants to environmental factors (Sardans et al., 2012). Warming and N deposition could affect the leaf N dynamics of plants (Zhang et al., 2017). In general, leaf N content is always positively correlated with plant photosynthetic capacity because most of leaf N constitutes photosynthetic enzymes and chlorophyll (Fu et al., 2015). In our study, we observed that warming significantly reduced plant N accumulation, and this is similar to Sardans et al.'s (2008) findings. Leaf N content is generally closely related with photosynthetic carbon fixation (Sardans et al., 2008). Previous researchers reported that warming can promote CO_2_ fixation in high latitude ecosystems, as temperature there might be far from optimum (Keeling et al., 1996). However, warming might also limit photosynthetic capacity for decrease of soil moisture, inducing negative effects on CO_2_ assimilation (Xu et al., 2014). Our results suggested that warming can decrease plant leaf N level for biochemistry process. Leaf N content is always enhanced by N deposition for higher soil N availability (Hobbie et al., 2001). N deposition can significantly increase plant N concentration and decrease C/N ratio (Esmeijer-Liu et al., 2009). These findings are in agreement with our results. Additionally, we found that N deposition can regulate the warming-induced impact on leaf N content.

Chlorophyll is of significant importance in determining plant photosynthesis (Zhang et al., 2016).

Some scholars have found that warming could cause no obvious impact on plant chlorophyll concentrations (Ren et al., 2010). In this study, we found that warming significantly decreased the chlorophyll content of *L. secalimus*. This is in agreement with Shen et al.'s (2013) findings. Nitrogen deposition induced a significant increase in the chlorophyll contents of *L. secalimus*. This is consistent with (Zhou et al., 2011) results. The possible reason might be that N deposition can further increase soil N availability and promote plant N uptake, leading to enhanced photosynthetic pigment synthesis (Zhang et al., 2013). Additionally, increase of chlorophyll suggested that N deposition could also promote photosynthesis (Zhang et al., 2016). The combined effects of warming and N deposition also significantly promoted the synthesis of photosynthetic pigments, while the impacts were not as stronger as warming alone. These results suggested that combined effects of warming and N deposition on plant photosynthetic pigments are still positive, though warming may limit the process of photosynthetic pigments synthesis.

### Warming and N Deposition Have Different Effects on the Antioxidant System and Photosynthetic Enzyme

Antioxidant enzyme activity is an important defense mechanism that influences stress tolerance in plants (Naudts et al., 2014). It is well known that reactive oxygen species could cause lipid peroxidation and impact cell membrane (Foyer et al., 1994). Membrane lipid peroxidation can reflect stress induced accumulation of reactive oxygen species (ROS) and produce malondialdehyde (MDA), which is widely used as an indicator of membrane injury (Zhou et al., 2018). We found in this study that warming significantly increased the MDA contents of *L. secalimus*, while N deposition alone decreased the MDA contents, indicating that warming can cause plasma membrane damage in *L. secalimus*. N deposition may enhance plant ROS scavenging capacity, and thus alleviate exterior stress on plant plasma membrane damage. Plants have developed a protective enzyme system as defense against oxygen toxicity under stress, such as SOD, POD, and CAT (Zhao and Liu, 2009). Our results suggested that warming led to a much higher antioxidant activity in *L. secalimus*, indicating that warming under stress can significantly motivate the antioxidant enzyme system. Nitrogen could promote synthesis of physiological activities of antioxidant enzymes (Zhao and Liu, 2009). Under warming plus N deposition, the activity of antioxidant enzymes was much lower than that under warming alone; this might be attributed to the fact that N deposition could alleviate the negative effects induced by warming to some degree.

### Main Path Determining the Change of *A_net_* Under Warming and N Deposition

Warming may cause water deficit and reduced stomatal conductance, which will result in lower *C_i_*, and consequently lead to a decrease in CO_2_ concentration at the carboxylation site of ribulose-1,5-bisphosphate (RuBP) carboxylase/oxygenase (Rubisco), thereby decreasing net photosynthesis (Chastain et al., 2014). *g_s_* was usually used as an indicator to evaluate stomatal and non-stomatal limitations to photosynthesis under water deficit (Brodribb, 1996). In our study, warming directly restrained *A_net_* by reducing the *g_s_* and Chl, suggesting that both stomatal limitation and non-stomatal limitation play an important role in determining the decrease of photosynthetic capacity under warming, and the downregulation of photosynthesis can result from lower CO_2_ availability caused by stomatal closure and the lower synthesis of Chl. In addition, though N deposition significantly increased *g_s_*, Chl content, and Rubisco activity, it directly increased *A_net_* by increasing the Chl content and Rubisco activity, indicating that non-stomatal limitation plays a vital role in determining the increase of photosynthetic capacity under N deposition.

N deposition could regulate plant responses to warming (Zong et al., 2018b), and change in temperature is also likely to interact with the effects of N deposition (Suddick et al., 2013). Up to now, little is known about the physiological responses of plants under warming and N deposition.

## Conclusion

Our study showed that climate warming can cause impact on photosynthesis of *L. secalimus*, while N deposition could induce positive effects. Warming reduced plant photosynthetic capacity mainly through both stomatal limitation and non-stomatal limitation. N deposition enhanced plant photosynthetic capacity mainly through non-stomatal limitation. Additionally, both warming and N deposition enhanced plant resistance by improving antioxidant enzyme systems. N deposition is beneficial for it can significantly increase stomatal conductance, chlorophyll content, and Rubisco activity, which are positively correlated with photosynthesis. Overall, warming could impair the photosynthetic potential of *L. secalinus* enhanced by N deposition in alpine grassland of Qinghai- Tibetan plateau. Future researches are needed to explore the causes of the different responses of *L. secalimus*, the dominant plant species of alpine grasslands, to the combination of climate warming and N deposition.

## Data Availability Statement

The datasets generated for this study are available on request to the corresponding author. 

## Author Contributions

SD, HS planned and designed the research. SD, WW, SLi, JX, MY, JZ, XG, YX and YZ helped to perform the experiments. SD, WW, SLiu, QD and HZ helped to give some useful suggestions in writing. SD, WW, HS, JY revised this manuscript. HS analyzed the data and wrote this paper.

## Funding

This research was financially supported by the grants from the National Key R & D Program of China (2016YFC0501906), Qinghai Provincial Key R & D program in Qinghai Province (2019-SF-145 & 2018-NK-A2), and State Key Joint Laboratory of Environmental Simulation and Pollution Control (Beijing Normal University) (17L03ESP). The authors would also thank the anonymous reviewers for their helpful comments; the endeavor of editors and reviewers was also appreciated.

## Conflict of Interest

The authors declare that the research was conducted in the absence of any commercial or financial relationships that could be construed as a potential conflict of interest.

## Abbreviations

*A_net_*, net photosynthetic rate; *g_s_*, stomatal conductance; *C_i_*, intercellular CO_2_ concentration; *T_r_*, transpiration rate; *VPD*, Vapor pressure deficit; *L_s_*, stomatal limitation; *Chla*, Chlorophyll a; *Chlb*, Chlorophyll b; *Chl*, total Chlorophyll; *MDA*, malondialdehyde; *SOD*, superoxide dismutase; *POD*, peroxidase; *CAT*, catalase; Rubisco, ribulose-1,5-bisphosphate carboxylase/oxygenase; *Fv*/*Fm*, maximal quantum yield of PSII; *ΦPSII*, effective quantum yield of PSII; *qP*, photochemical quenching; *NPQ*, non-photochemical quenching; FW, fresh weight.
